# 1-Bromo­adamantane

**DOI:** 10.1107/S1600536808038452

**Published:** 2008-12-13

**Authors:** Richard Betz, Peter Klüfers, Peter Mayer

**Affiliations:** a Ludwig-Maximilians Universität, Department Chemie und Biochemie, Butenandtstrasse 5–13 (Haus D), 81377 München, Germany

## Abstract

The mol­ecule of the title compound, C_10_H_15_Br, shows noncrystallographic mirror symmetry. In the crystal structure, no inter­molecular inter­actions with distances less than the sum of the van der Waals radii of the respective atoms are present.

## Related literature

For the crystal structure of the thio­urea solvate of the compound, see Chao *et al.* (2003 ▶).
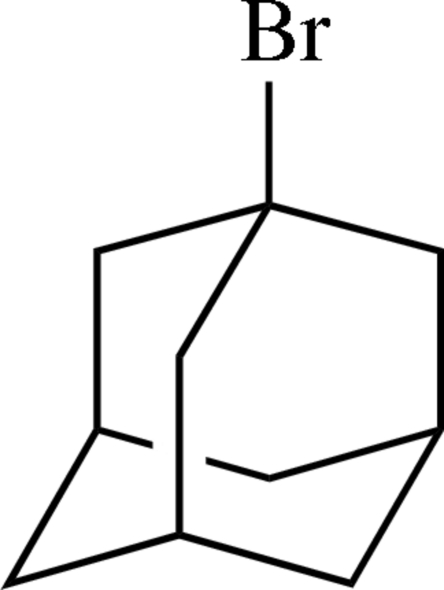


## Experimental

### 

#### Crystal data

C_10_H_15_Br
*M* *_r_* = 215.13
Monoclinic, 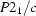

*a* = 10.154 (3) Å
*b* = 6.8541 (11) Å
*c* = 13.240 (3) Å
β = 90.027 (17)°
*V* = 921.5 (4) Å^3^
*Z* = 4
Mo *K*α radiation
μ = 4.40 mm^−1^
*T* = 200 (2) K
0.21 × 0.16 × 0.13 mm

#### Data collection

Oxford Xcalibur diffractometer
Absorption correction: analytical (de Meulenaer & Tompa, 1965 ▶) *T*_min_ = 0.462, *T*_max_ = 0.614
4563 measured reflections
1629 independent reflections
1313 reflections with *I* > 2σ(*I*)
*R*_int_ = 0.054

#### Refinement

*R*[*F* ^2^ > 2σ(*F* ^2^)] = 0.032
*wR*(*F* ^2^) = 0.080
*S* = 1.02
1629 reflections
101 parameters
H-atom parameters constrained
Δρ_max_ = 0.77 e Å^−3^
Δρ_min_ = −0.38 e Å^−3^

### 

Data collection: *CrysAlis CCD* (Oxford Diffraction, 2005 ▶); cell refinement: *CrysAlis RED* (Oxford Diffraction, 2005 ▶); data reduction: *CrysAlis RED*; program(s) used to solve structure: *SHELXS97* (Sheldrick, 2008 ▶); program(s) used to refine structure: *SHELXL97* (Sheldrick, 2008 ▶); molecular graphics: *ORTEP-3* (Farrugia, 1997 ▶); software used to prepare material for publication: *SHELXL97*.

## Supplementary Material

Crystal structure: contains datablocks global, I. DOI: 10.1107/S1600536808038452/hg2442sup1.cif

Structure factors: contains datablocks I. DOI: 10.1107/S1600536808038452/hg2442Isup2.hkl

Additional supplementary materials:  crystallographic information; 3D view; checkCIF report

## Figures and Tables

**Fig. 1.**
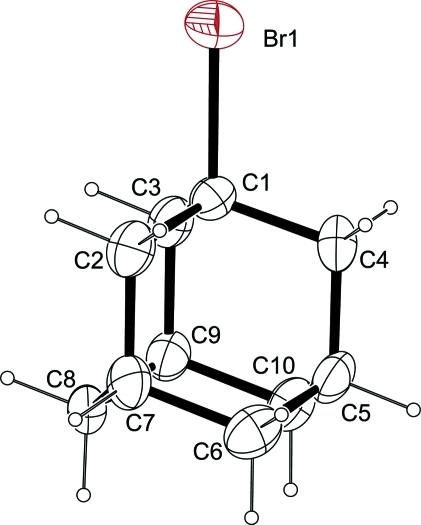
The molecular structure of the title compound, with atom labels and anisotropic displacement ellipsoids (drawn at 50% probability level) for non-H atoms.

**Fig. 2.**
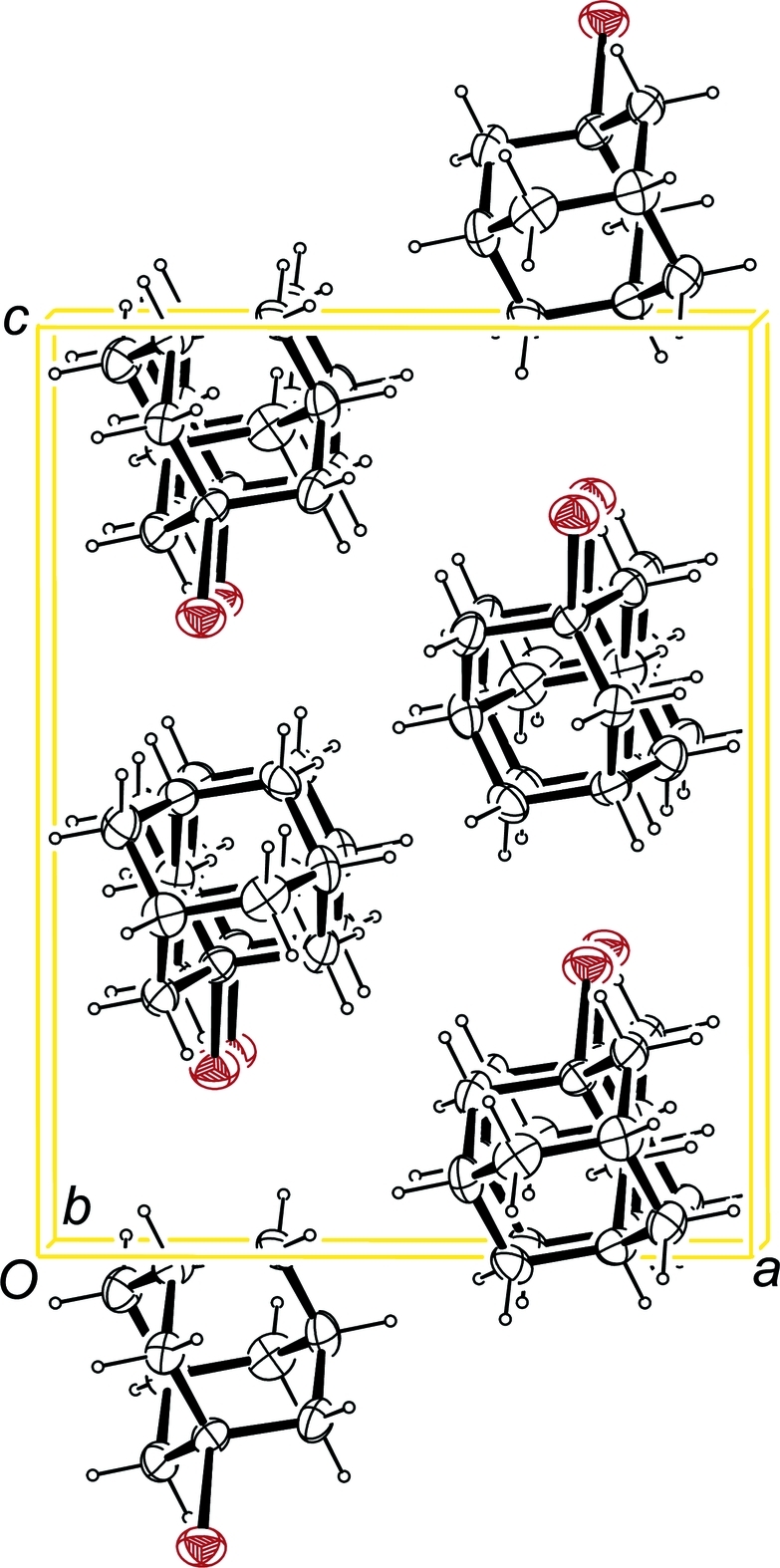
The packing of the title compound, viewed along [010].

## supplementary crystallographic information

### Comment

The structure of the title compound was elucidated for comparison of the influence of different substituents on the geometry of the adamantane framework.

In the molecule the Br atom is bonded to one of the bridgehead positions of the carbocycle (Fig. 1). Bond lengths are normal.

In the crystal structure, only dispersive interactions are present. No intermolecular contacts whose range falls below the sum of the van der Waals radii of the respective atoms are existent.

A similar structure, the thiourea solvate of the compound, has been described by Chao *et al.* (2003) but showed disorder among the 1-bromoadamantane moiety. However, a comparison of both molecules shows good agreement in terms of bond lengths and angles.

The packing of the compound is shown in Fig. 2.

### Experimental

The compound was obtained commercially (ACROS). Crystals suitable for X-ray analysis were obtained upon free evaporation of a solution of the compound in diethyl ether.

### Refinement

Carbon-bound H-atoms were placed in calculated positions (C—H 0.99 Å for methylene groups and C—H 1.00 Å for bridgehead positions) and were included in the refinement in the riding model approximation, with *U*(H) set to 1.2*U*_eq_(C).

The crystal measured is refined as a twin with a twin-plane perpendicular to [001] (*Ebenenzwilling*). The volume-to-volume-ratio for the two individuals is found at approximately 1:1 with a batch-scale factor of approximately 0.46.

### Crystal data

**Table:**

C_10_H_15_Br	*F*(000) = 440
*M**_r_* = 215.13	*D*_x_ = 1.551 Mg m^−^^3^
Monoclinic, *P*2_1_/*c*	Mo *K*α radiation, λ = 0.71073 Å
Hall symbol: -P 2ybc	Cell parameters from 2345 reflections
*a* = 10.154 (3) Å	θ = 3.9–26.3°
*b* = 6.8541 (11) Å	µ = 4.39 mm^−^^1^
*c* = 13.240 (3) Å	*T* = 200 K
β = 90.027 (17)°	Block, colourless
*V* = 921.5 (4) Å^3^	0.21 × 0.16 × 0.13 mm
*Z* = 4	

### Data collection

**Table:**

Oxford Xcalibur diffractometer	1629 independent reflections
Radiation source: fine-focus sealed tube	1313 reflections with *I* > 2σ(*I*)
graphite	*R*_int_ = 0.054
ω scans	θ_max_ = 25.3°, θ_min_ = 3.9°
Absorption correction: analytical (de Meulenaer & Tompa, 1965)	*h* = −12→11
*T*_min_ = 0.462, *T*_max_ = 0.614	*k* = −8→8
4563 measured reflections	*l* = −15→14

### Refinement

**Table:**

Refinement on *F*^2^	Primary atom site location: structure-invariant direct methods
Least-squares matrix: full	Secondary atom site location: difference Fourier map
*R*[*F*^2^ > 2σ(*F*^2^)] = 0.032	Hydrogen site location: inferred from neighbouring sites
*wR*(*F*^2^) = 0.080	H-atom parameters constrained
*S* = 1.02	*w* = 1/[σ^2^(*F*_o_^2^) + (0.0393*P*)^2^] where *P* = (*F*_o_^2^ + 2*F*_c_^2^)/3
1629 reflections	(Δ/σ)_max_ < 0.001
101 parameters	Δρ_max_ = 0.77 e Å^−^^3^
0 restraints	Δρ_min_ = −0.37 e Å^−^^3^

### Fractional atomic coordinates and isotropic or equivalent isotropic displacement parameters (Å^2^)

**Table:**

	*x*	*y*	*z*	*U*_iso_*/*U*_eq_	
Br1	0.23244 (6)	0.64478 (6)	0.19046 (4)	0.04391 (18)	
C1	0.2412 (4)	0.4548 (6)	0.3057 (3)	0.0281 (9)	
C2	0.3872 (4)	0.4075 (6)	0.3225 (4)	0.0361 (11)	
H21	0.4369	0.5279	0.3386	0.043*	
H22	0.4255	0.3481	0.2610	0.043*	
C3	0.1789 (4)	0.5508 (7)	0.3973 (3)	0.0315 (11)	
H31	0.0859	0.5840	0.3829	0.038*	
H32	0.2266	0.6724	0.4144	0.038*	
C4	0.1651 (5)	0.2707 (7)	0.2754 (3)	0.0330 (11)	
H41	0.2042	0.2120	0.2140	0.040*	
H42	0.0719	0.3031	0.2612	0.040*	
C5	0.1743 (6)	0.1266 (7)	0.3651 (4)	0.0356 (14)	
H5	0.1251	0.0048	0.3477	0.043*	
C6	0.3174 (6)	0.0759 (8)	0.3849 (5)	0.0418 (15)	
H61	0.3563	0.0147	0.3241	0.050*	
H62	0.3233	−0.0185	0.4413	0.050*	
C7	0.3940 (4)	0.2628 (8)	0.4118 (4)	0.0372 (13)	
H7	0.4880	0.2294	0.4261	0.045*	
C8	0.3321 (6)	0.3549 (7)	0.5051 (4)	0.0345 (14)	
H81	0.3807	0.4752	0.5229	0.041*	
H82	0.3390	0.2633	0.5627	0.041*	
C9	0.1869 (6)	0.4047 (7)	0.4865 (4)	0.0317 (13)	
H9	0.1480	0.4648	0.5486	0.038*	
C10	0.1118 (5)	0.2199 (8)	0.4592 (4)	0.0369 (13)	
H101	0.0184	0.2519	0.4454	0.044*	
H102	0.1149	0.1268	0.5163	0.044*	

### Atomic displacement parameters (Å^2^)

**Table:**

	*U*^11^	*U*^22^	*U*^33^	*U*^12^	*U*^13^	*U*^23^
Br1	0.0506 (3)	0.0472 (3)	0.0339 (2)	−0.0028 (3)	−0.0007 (3)	0.0092 (2)
C1	0.030 (2)	0.0264 (19)	0.0282 (19)	−0.0018 (18)	0.003 (4)	0.0013 (18)
C2	0.027 (2)	0.040 (3)	0.042 (3)	−0.006 (2)	0.007 (2)	−0.002 (2)
C3	0.031 (3)	0.026 (2)	0.037 (3)	0.000 (2)	0.001 (2)	−0.005 (2)
C4	0.029 (2)	0.037 (3)	0.033 (2)	−0.008 (2)	−0.001 (2)	−0.009 (2)
C5	0.042 (3)	0.023 (3)	0.042 (3)	−0.010 (2)	0.000 (3)	−0.003 (2)
C6	0.050 (4)	0.029 (3)	0.046 (3)	0.001 (3)	0.006 (3)	−0.002 (3)
C7	0.024 (2)	0.040 (3)	0.048 (4)	−0.006 (3)	−0.005 (2)	0.002 (3)
C8	0.035 (3)	0.036 (3)	0.033 (3)	−0.012 (3)	−0.009 (2)	0.002 (2)
C9	0.036 (3)	0.031 (3)	0.029 (3)	−0.007 (2)	0.003 (2)	−0.003 (2)
C10	0.033 (3)	0.035 (3)	0.043 (3)	−0.008 (2)	0.004 (3)	0.001 (2)

### Geometric parameters (Å, °)

**Table:**

Br1—C1	2.008 (4)	C5—C10	1.538 (8)
C1—C3	1.518 (6)	C5—H5	1.0000
C1—C4	1.533 (6)	C6—C7	1.540 (8)
C1—C2	1.533 (6)	C6—H61	0.9900
C2—C7	1.545 (7)	C6—H62	0.9900
C2—H21	0.9900	C7—C8	1.523 (8)
C2—H22	0.9900	C7—H7	1.0000
C3—C9	1.551 (6)	C8—C9	1.533 (7)
C3—H31	0.9900	C8—H81	0.9900
C3—H32	0.9900	C8—H82	0.9900
C4—C5	1.547 (7)	C9—C10	1.522 (7)
C4—H41	0.9900	C9—H9	1.0000
C4—H42	0.9900	C10—H101	0.9900
C5—C6	1.517 (8)	C10—H102	0.9900
			
C3—C1—C4	110.8 (4)	C5—C6—C7	109.4 (5)
C3—C1—C2	112.3 (4)	C5—C6—H61	109.8
C4—C1—C2	110.6 (4)	C7—C6—H61	109.8
C3—C1—Br1	107.9 (3)	C5—C6—H62	109.8
C4—C1—Br1	108.2 (3)	C7—C6—H62	109.8
C2—C1—Br1	106.9 (3)	H61—C6—H62	108.2
C1—C2—C7	106.8 (4)	C8—C7—C6	108.9 (4)
C1—C2—H21	110.4	C8—C7—C2	109.6 (4)
C7—C2—H21	110.4	C6—C7—C2	109.6 (4)
C1—C2—H22	110.4	C8—C7—H7	109.6
C7—C2—H22	110.4	C6—C7—H7	109.6
H21—C2—H22	108.6	C2—C7—H7	109.6
C1—C3—C9	107.9 (4)	C7—C8—C9	111.1 (5)
C1—C3—H31	110.1	C7—C8—H81	109.4
C9—C3—H31	110.1	C9—C8—H81	109.4
C1—C3—H32	110.1	C7—C8—H82	109.4
C9—C3—H32	110.1	C9—C8—H82	109.4
H31—C3—H32	108.4	H81—C8—H82	108.0
C1—C4—C5	107.1 (4)	C10—C9—C8	109.5 (5)
C1—C4—H41	110.3	C10—C9—C3	109.3 (4)
C5—C4—H41	110.3	C8—C9—C3	108.4 (4)
C1—C4—H42	110.3	C10—C9—H9	109.9
C5—C4—H42	110.3	C8—C9—H9	109.9
H41—C4—H42	108.5	C3—C9—H9	109.9
C6—C5—C10	110.5 (5)	C9—C10—C5	109.4 (4)
C6—C5—C4	109.7 (5)	C9—C10—H101	109.8
C10—C5—C4	109.4 (4)	C5—C10—H101	109.8
C6—C5—H5	109.1	C9—C10—H102	109.8
C10—C5—H5	109.1	C5—C10—H102	109.8
C4—C5—H5	109.1	H101—C10—H102	108.2
			
C3—C1—C2—C7	61.4 (5)	C5—C6—C7—C2	−60.8 (6)
C4—C1—C2—C7	−62.9 (5)	C1—C2—C7—C8	−58.8 (5)
Br1—C1—C2—C7	179.5 (3)	C1—C2—C7—C6	60.6 (5)
C4—C1—C3—C9	62.2 (5)	C6—C7—C8—C9	−59.1 (6)
C2—C1—C3—C9	−62.0 (5)	C2—C7—C8—C9	60.7 (5)
Br1—C1—C3—C9	−179.5 (3)	C7—C8—C9—C10	59.1 (5)
C3—C1—C4—C5	−62.2 (5)	C7—C8—C9—C3	−60.0 (5)
C2—C1—C4—C5	62.9 (5)	C1—C3—C9—C10	−60.4 (5)
Br1—C1—C4—C5	179.6 (3)	C1—C3—C9—C8	58.9 (5)
C1—C4—C5—C6	−60.9 (6)	C8—C9—C10—C5	−58.0 (6)
C1—C4—C5—C10	60.5 (5)	C3—C9—C10—C5	60.6 (5)
C10—C5—C6—C7	−60.0 (6)	C6—C5—C10—C9	59.7 (6)
C4—C5—C6—C7	60.7 (6)	C4—C5—C10—C9	−61.2 (5)
C5—C6—C7—C8	59.1 (6)		
